# A cognitive deep learning approach for medical image processing

**DOI:** 10.1038/s41598-024-55061-1

**Published:** 2024-02-24

**Authors:** Hussam N. Fakhouri, Sadi Alawadi, Feras M. Awaysheh, Fahed Alkhabbas, Jamal Zraqou

**Affiliations:** 1 Department of Data Science and Artificial Intelligence, The University of Petra, Amman, Jordan; 2 Department of Computer Science, Blekinge Institute of Technology, Karlskrona, Sweden; 3 Computer Graphics and Data Engineering (COGRADE) Research Group, University of Santiago de Compostela, Santiago de Compostela, Spain; 4 Institute of Computer Science, Delta Research Centre, University of Tartu, Tartu, Estonia; 5 Internet of Things and People Research Center, Malmö University, Malmö, Sweden; 6 Department of Computer Science and Media Technology, Malmö University, Malmö, Sweden; 7 Virtual and Augment Reality Department, Faculty of Information Technology, University of Petra, Amman, Jordan

**Keywords:** Prognosis, Computer science, Information technology, Scientific data

## Abstract

In ophthalmic diagnostics, achieving precise segmentation of retinal blood vessels is a critical yet challenging task, primarily due to the complex nature of retinal images. The intricacies of these images often hinder the accuracy and efficiency of segmentation processes. To overcome these challenges, we introduce the cognitive DL retinal blood vessel segmentation (CoDLRBVS), a novel hybrid model that synergistically combines the deep learning capabilities of the U-Net architecture with a suite of advanced image processing techniques. This model uniquely integrates a preprocessing phase using a matched filter (MF) for feature enhancement and a post-processing phase employing morphological techniques (MT) for refining the segmentation output. Also, the model incorporates multi-scale line detection and scale space methods to enhance its segmentation capabilities. Hence, CoDLRBVS leverages the strengths of these combined approaches within the cognitive computing framework, endowing the system with human-like adaptability and reasoning. This strategic integration enables the model to emphasize blood vessels, accurately segment effectively, and proficiently detect vessels of varying sizes. CoDLRBVS achieves a notable mean accuracy of 96.7%, precision of 96.9%, sensitivity of 99.3%, and specificity of 80.4% across all of the studied datasets, including DRIVE, STARE, HRF, retinal blood vessel and Chase-DB1. CoDLRBVS has been compared with different models, and the resulting metrics surpass the compared models and establish a new benchmark in retinal vessel segmentation. The success of CoDLRBVS underscores its significant potential in advancing medical image processing, particularly in the realm of retinal blood vessel segmentation.

## Introduction

Artificial intelligence (AI) and cognitive computing have revolutionized various sectors, including medicine, pharmacy, and healthcare, in the realm of big data^1^. While AI operates on algorithms and patterns, cognitive computing takes it further, mimicking the human brain’s reasoning processes and adaptability to deliver more nuanced solutions. Together, their transformative power is redefining modern medical image processing, streamlining processes, enhancing accuracy, and potentially saving lives. The synergy between AI, cognitive computing, and medical imaging offers robust tools that extend and amplify human capabilities in diagnosing and treating various diseases^2^.

In this regard, recent medical imaging technology advancements allow us to capture retina details with unprecedented clarity^3^. Nevertheless, research is still seeking more accuracy and efficiency due to the domain’s natural complexities. For instance, retinal blood vessel segmentation demands precise differentiation of these vessels, which presents numerous challenges^4^. Traditional manual and semi-automatic methods, plagued by inefficiency and proneness to errors, falter, especially when faced with the voluminous data churned out by modern imaging systems^5^.

The convergence of AI’s algorithmic and cognitive computing ushers a transformative shift using the amalgamation of hybrid models. With the capacity to learn, adapt, and discern complex patterns from vast datasets, this confluence is reshaping healthcare^6^. Tasks like image segmentation, disease identification, and prognosis prediction have recently seen a significant infusion of AI and cognitive computing principles. AI, specially trained on extensive retinal image datasets, can refine its accuracy in blood vessel delineation, converting raw data into actionable insights^7^. Also, convolutional neural networks (CNNs) stand out for their adeptness in image analysis due to their hierarchical data learning^6^.

Deep learning, enriched with cognitive approaches, can surpass traditional methods in accuracy and efficiency for retinal blood vessel segmentation^8^. However, exploring the full capability of combining AI and cognitive computing in diagnostics and mitigating vision loss is still far from its potential. Various deep learning architectures have been used for retinal blood vessel segmentation, such as convolutional neural networks (CNNs)^9^, fully convolutional networks (FCNs)^10^, and U-nets^11^. These architectures are trained on different datasets of retinal images labeled with the location of blood vessels, allowing them to learn to identify the vessels in new images^8^ accurately. Automatic analysis of the retinal vascular tree by image processing techniques is essential for many clinical investigations and constitutes a field of scientific research leading^12^. Detection and characterization of small blood vessels on retina images are essential in diagnosing certain diseases, such as diabetes or hypertension. The current state of the art indicates that methods based on supervised learning currently have the best performance. However, accurate segmentation of retinal blood vessels is a crucial step in diagnosing and monitoring various ocular diseases like diabetic retinopathy and glaucoma.

Nevertheless, the task is challenging due to the intricate and varying structure of the retinal blood vessels, the presence of pathologies, and differences in image quality. While demonstrating promise, current methods still need to be revised regarding sensitivity, specificity, and robustness to image variations. Dash et al.^13^ highlights a pressing need for an advanced, reliable, and more precise retinal blood vessel segmentation technique to address these challenges. Thus, this research proposes a new hybrid model for retinal blood segmentation and tries to answer the following questions.

This research aims to address the previous concern and propose an innovative approach called CoDLRBVS to improve the detection and segmentation of fine retinal vessels by integrating deep learning methods(i.e., U-Net architecture) with diverse image processing techniques, such as matched filter (MF), multi-scale line detection, scale space representation, and morphological operations. Each technique contributes to creating an approach that emulates human cognition and adaptability in processing complex visual information such as medical images. In our proposed approach, the matched filter and multi-scale line detection are calibrated to amplify the initial segmentation of blood vessels, integrating cognitive principles to interpret intricate patterns and enhance contrasting features, much like the human visual system. Subsequently, the U-Net architecture, revered for its efficacy in biomedical image segmentation, refines these results by learning hierarchical features and making informed decisions, reflecting human-like analytical reasoning.

Moreover, scale space representation is implemented to analyze blood vessels at varied scales, mirroring the human ability to perceive objects at different distances and sizes, and morphological operations are employed to refine the segmentation results by eliminating noise and filling gaps, emulating the human brain’s inherent ability to filter out irrelevant information and focus on the essential. The culmination of these techniques is expected to yield a system that transcends the current paradigms in segmentation methods regarding accuracy, sensitivity, and specificity, facilitating early detection and intervention for retinal diseases. Hence, CoDLRBVS provides a unique integration of a pre-processing phase using the MF for feature enhancement. Also, CoDLRBVS includes a post-processing phase employing morphological techniques (MT) to refine the segmentation output. Finally, it incorporates multi-scale line detection and scale space methods to enhance segmentation capabilities.

The main contribution of this paper is CoDLRBVS, an innovative approach to improve the detection and segmentation of fine retinal vessels. Compared to our approach, most existing approaches fail to handle retinal images’ intrinsic variability and complexity. CoDLRBVS integrates deep learning methods, mainly U-Net architecture, and diverse image processing techniques to address such shortcomings and achieve better results. The developed model exhibits robust performance across different retinal image datasets and under various image quality conditions, as proved by our experiments. CoDLRBVS achieved high segmentation performance with a mean accuracy of 96.7% across all datasets. Additionally, the model supports the adaptation to various sizes of retinal blood vessels by effectively incorporating adaptive techniques like scale space and multi-scale line detection into the U-Net architecture.

The remainder of this paper is organized as follows: “Background and related work” provides background on retinal imaging, cognitive computing, image pre-processing techniques, and convolutional neural networks and explores the related work in the field. Next, we introduce the proposed approach and its main phases in “CoDLRBVS approach”. The datasets used in the study, experimental settings, approach evaluation, and the obtained results are described in “Result analysis and experiment description”. Finally, we conclude the paper in “Conclusion”, draw the main finding, and outline potential future work direction.

## Background and related work

In this section, we present background about closely related fields, including retinal imaging, cognitive computing, and image pre-processing techniques in medical image processing. Additionally, we discuss studies related to our work.

### Background

#### Retinal imaging

Ophthalmologists rely on retinal imaging because it enables them to diagnose and treat eye disorders at an early stage. Retinal imaging plays a crucial role in health prediction enabled by deep learning techniques^14^. There are multiple types of retinal imaging methods, including the following ones^14^: (1) fundus photography. It is the most frequent form of retinal imaging, which results in a color picture of the retina. Its primary applications are in the early detection and follow-up of retinal disorders. (2) optical coherence tomography. It produces cross-sectional images of the retina, enabling the measurement of retinal thickness and the identification of minor structural changes that may not be detectable with conventional imaging techniques. The macular hole, macular pucker, and macular edema are all disorders that benefit greatly from this diagnosis and treatment method. (3) Adaptive optics in imaging. This cutting-edge method provides real-time correction for optical flaws in the eye and results in cellular-level retina imaging.

#### Cognitive computing

It simulates human thinking processes in computers, employs self-learning algorithms for utilizing data mining, pattern recognition, and natural language processing. Gudivada et al.^15^ provided insights into its relevance in diagnosis assistance, drug discovery, and patient management. The authors emphasized its potency in processing complex medical imaging datasets, making it suitable for retinal vessel segmentation.

Beyond data analysis, cognitive computing holds transformative potential for patient care, research, and healthcare operations in many areas, including RNA sequencing^16,17^. Srivani et al.^18^ illustrated its pivotal role in patient-centric care, spotlighting its capability in predicting patient needs and shaping care plans, especially in managing chronic diseases. Similarly, Kumar et al.^19^ elaborated on system’s efficacy in adaptability in dynamic healthcare settings for predicting patient inflow, optimizing hospital resources, staffing, and bed allocation. Sathananthavathi and Indumathi showcased potential of retinal imaging in^20,21^. In^20^, the authors integrated cognitive systems with image processing for retinal vessel segmentation. Whereas in^21^, the authors utilized cognitive computing to discern anomalies in retinal images linked to diabetic retinopathy, highlighting the adaptive evolution of cognitive models for improved accuracy over time.

Deep learning techniques, especially CNNs, have made remarkable strides in medical imaging. With recent progress in CNN architectures, there has been a surge in precise image segmentation. For instance, Chen et al.^22^ enhanced the U-Net structure tailored for retinal blood vessel segmentation by integrating with conventional image processing. Traditionally, vessel segmentation has utilized techniques like adaptive thresholding and morphological operations and notably, the outcomes were enhanced when integrated with machine learning techniques. Wang et al.^23^ demonstrated innovative use of cognitive systems with generative adversarial networks for medical image augmentation. Similarly, Nahiduzzaman et al.^24^ illustrated the benefits of integrating cognitive systems with CNNs in chest X-ray imaging, using cognition to understand anomalies and direct the CNNs more accurately. Furthermore, Shrma et al.^25^ spotlighted the advantages of integrating cognitive systems and neural architectures in ultrasound imagery, demonstrating cognitive systems’ potential in aiding deep learning models to discern ambiguous regions, and consequently honing segmentation and classification.

#### Image pre-processing techniques

Image pre-processing techniques play prominent role in enhancing the quality of the images, which is crucial for accurate diagnosis and treatment planning^26,27^. The normalization techniques enable the adjustment of pixel intensity of medical images to a standard range, resulting in improving the contrast and making the details more visible. This technique is useful in medical scenarios where images suffer from poor contrast mainly due to the imaging environment^28^. It exploits linear or non-linear adjustments to improve images’ clarity for medical analysis purposes^29^.

Color space conversion is another important pre-processing step in medical imaging. This step is important in scenarios where color information is crucial, such as in histology images or stained tissue samples^30^. Converting images into appropriate color spaces (e.g., from RGB to the CIE Lab* color space) would enable medical professionals to study specific features more effectively^31^. Moreover, matched filters is another technique applied in the medical imaging to enhance specific patterns, such as the detection of microcalcifications in mammograms or blood vessels in retinal images. Using the cross-correlation between the image and a predefined pattern, matched filters can suppress the noise and detect and spotlight areas of interest^32^.

To identify and analyze structures of various sizes and orientations in medical images (e.g., such as blood vessels, neural pathways, or skeletal structures), multi-scale line detection technique cab be applied^33^. For this purpose, this technique involves examining images at multiple scales or resolutions^34^. This technique is useful in complex and critical medical scenarios such as detecting tumors and classifying tissues^35^.

#### Convolutional neural networks

CNN is a class of deep learning algorithms designed to automatically and adaptively learn spatial hierarchies of features. Thus, CNN are suitable for analyzing visual data^36^. U-Net is one of the most notable CNN architectures for medical image segmentation^11^. The U-Net is useful to achieve more accurate segmentations in case less training data is available. It is commonly used in fields, including magnetic resonance imaging, computed tomography, and microscopy. Other commonly used CNN architectures applied in the medical imaging domain include SegNet^37^, V-Net^38^, and DeepLab^39^.

### Related work

Soares et al.^40^ developed a supervised technique for the segmentation of vessels. The technique exploits a two-dimensional Gabor wavelet and a selection of morphological variables. The experiments results indicated that the technique was effective in separating the vessels from the backdrop. Staal et al.^41^ proposed an unsupervised and automated method for vessel segmentation. The method achieved reliable performance by utilizing a combination of line detectors and the hysteresis thresholding of the vessel’s likelihood map.

Dash et al.^42^ present a method to enhance the performance of curvelet transform. To improve retinal blood vessel segmentation, the method enables the fusion of curvelet transform and the Jerman filter, while the Mean-C threshold is used for the segmentation purpose. Further, in^43^, the authors developed an automated method to extract the blood vessels from fundus. For this purpose, the method integrates discrete wavelet transform and Tyler Coye algorithm. Additionally, the methods exploits the gamma correction to enhance the images contrast. Furthermore, in^44^, the authors proposed a model for enhancing abnormal retinal images containing low vessel contrasts. For this purpose, the proposed approach exploits both a fast guided filter and a matched filter for improving the performance measures for vessel extraction.

The current state of art indicates that methods based on supervised learning currently have the best performance^8^. Those methods are based on patches of real-size images and use CNNs and start with classifying each pixel of the image according to a fixed centered pixel neighborhood^11^. Unsupervised learning algorithms represent another approach to segment retinal vessels. They are capable of automatically segmenting vessels without requiring annotated training data. Frangi vesselness filter is a well-known unsupervised learning method designed to enhance vessel-like structures in images^45^. Fraz et al.^46^ developed an ensemble classification method that exploits a combination of boosting and random forests ML algorithms with a novel set of rotating invariant features. The method achieved an improved performance over the other state-of-the-art techniques. Recently, several deep learning-based methods have been introduced to address the issue of retinal vessel segmentation. Orlando et al.^47^ proposed deep learning based approach that exploits a U-Net architecture. The proposed model was trained end-to-end on a large dataset and showed superior performance compared to other techniques. Melinscak et al.^48^ developed an approach based on multi-scale line detection and used it for segmentation of retinal blood vessels. The authors validated their approach by conducting experiments on four publicly available datasets and reported competitive results.

Further, CNNs have been extensively applied in retinal blood vessel segmentation^49^. They are designed to automatically and adaptively learn spatial hierarchies of features, making them exceptionally suited for image recognition tasks. Their architecture, which allows for the simultaneous examination of several scales, improves the detection of both small and large blood vessels^45^. Furthermore, the FCNs have also been utilized for the same purpose^50^. The main advantage of applying FCNs is that they support end-to-end and pixels-to-pixels learning without the need for patch extraction and selection. Consequently, this makes the segmentation process more efficient^10^. The U-net architecture is specifically designed for biomedical image segmentation. This makes it one of the most successful implementations of FCNs^11^. The U-net architecture consists of two paths where the first is a contracting path that capture the context. Whereas, the second is a symmetric expanding path that enables precise localization. Consequently, this unique architecture enables an effective retinal blood vessel segmentation process.

Moreover, methods based on the integration of deep learning and classical image processing techniques have also been proposed. For instance, Li et al.^51^ developed a framework that integrates CNNs and scale-space theory for blood vessel segmentation. Similarly, Zhang et al.^52^ introduced the LCU-Net, a novel low-cost U-Net based approach for the environmental microorganism image segmentation task. The approach extends and improves the traditional U-Net architecture by integrating inception and concatenate operations, which enables it to address the single receptive field’s limitations and high memory cost. In^53^, the authors conducted a comprehensive review considering different techniques, including conventional multilayer perceptrons, convolutional neural networks, and visual transformers. The findings of the review highlights the critical role of neural networks in various applications, including environmental pollution control and disease prevention.

Chen et al.^54^ developed IL-MCAM, a framework for colorectal histopathology image classification. The framework exploits interactive learning and multi-channel attention mechanism to enhance the images’ classification accuracy. Li et al.^55^ conducted a comprehensive review of automated image analysis techniques. The study discusses the progression of ML techniques and their integration into whole-slide image analysis. Additionally, it outlines the developments and challenges in feature extraction, segmentation, and classification methods. Finally, the optimization of retinal blood vessel segmentation has recently been widely used in many fields^56,57^. It also has been used for tuning the hyper-parameter of deep learning algorithms^36^, which can further enhance the blood vessel model.

To summarize, although several approaches have been proposed to enable the automated retinal blood vessel segmentation, they often fall short in handling the intrinsic variability and complexity of retinal images. Many existing models lack the adaptability to accurately segment vessels of varying sizes and shapes, or they do not adequately address the issues of noise and fine structure preservation in the images. Moreover, the integration of advanced image processing techniques with deep learning methods has not been fully explored or optimized, leaving potential improvements in accuracy and efficiency untapped. The reliance on single-scale methods or non-adaptive techniques often results in suboptimal performance, particularly in the presence of pathological changes or varied image qualities. Furthermore, while some models demonstrate decent performance on specific datasets, their generalizability across different datasets and under diverse imaging conditions remains a significant challenge. In light of these gaps, we propose the CoDLRBVS model, a novel hybrid approach that combines the strengths of image processing and deep learning. Our method addresses these shortcomings by introducing a more adaptable, robust, and precise segmentation solution, setting a new standard for accuracy and performance in retinal blood vessel segmentation.

## CoDLRBVS approach

The CoDLRBVS introduces an innovative approach to improve the detection and segmentation of fine retinal vessels by integrating deep learning algorithms, precisely the U-Net architecture model, with various image processing techniques. These techniques include matched filter, multi-scale line detection, scale space, and morphological operations as shown in algorithm 1. While each method contributes distinct advantages, their combined utilization significantly influences the final output of the approach.

For instance, applying the matched filter significantly improves the visibility and contrast of blood vessel structures’ essential details by amplifying the visibility of the vessels’ patterns and enhancing their detectability. The multi-scale line detection method allows the model to analyze the image at different scales. It ensures accurate detection and segmentation of vessels with various thicknesses that address the inherent diversity in vessel diameters. The U-Net model is designed explicitly for semantic segmentation tasks. Its encoder–decoder architecture enables it to capture local and global contextual information, which contributes to accurately segmenting the blood vessels.

**Figure 1:**
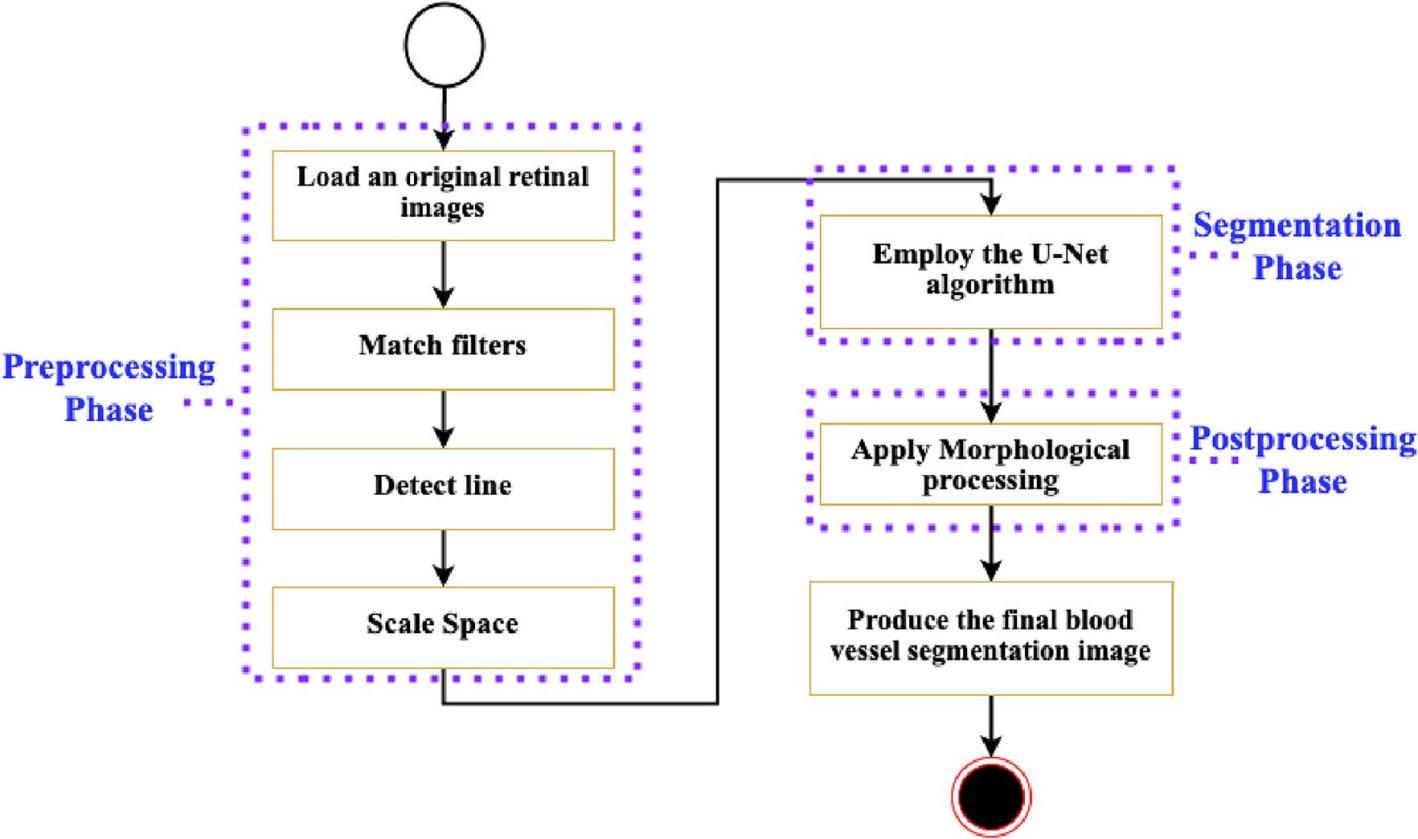
The CoDLRBVS steps to segment blood small vessel in retinal images.

Additionally, the scale-space analysis allows the model to effectively capture the vessel structures at distinct scales, considering both vessel size variations and thickness. Ultimately, leveraging morphological techniques refines the segmented vessel map by eliminating noise, filling gaps, enhancing both the overall connectivity and smoothness of the segmented vessels. These methods improve the CoDLRBVS’s robustness against noise, anomalies, and image variations, which are well-known issues in medical images.

**Algorithm 1:**
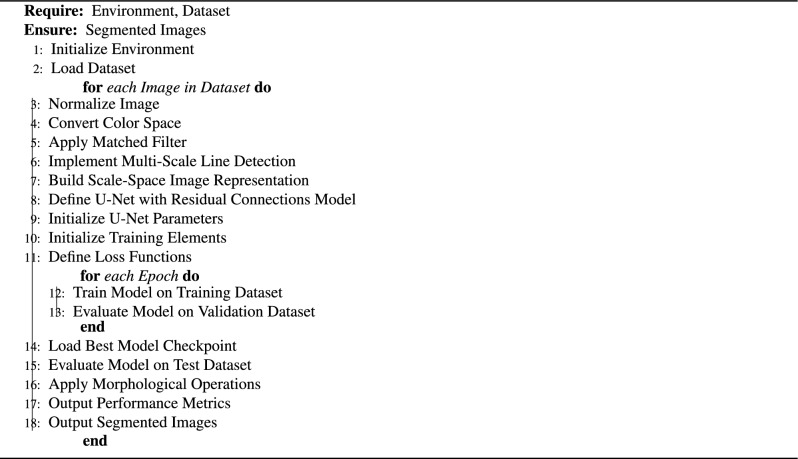
Retinal blood vessel segmentation using CoDLRBVS model.

By seamlessly incorporating these techniques with cognitive computing attributes, there is a notable increase in the accuracy and robustness of blood vessel segmentation. Moreover, the approach exhibits enhanced resilience against common challenges in medical images, such as noise and anomalies. This human-centred integrated approach holds transformative potential across various medical domains, spanning disease diagnosis, monitoring, surgical planning, and developing innovative treatment methodologies. Figure 1 illustrates the CoDLRBVS approach abstract diagram, while algorithm 1 shows the CoDLRBVS detailed steps.

**Figure 2:**
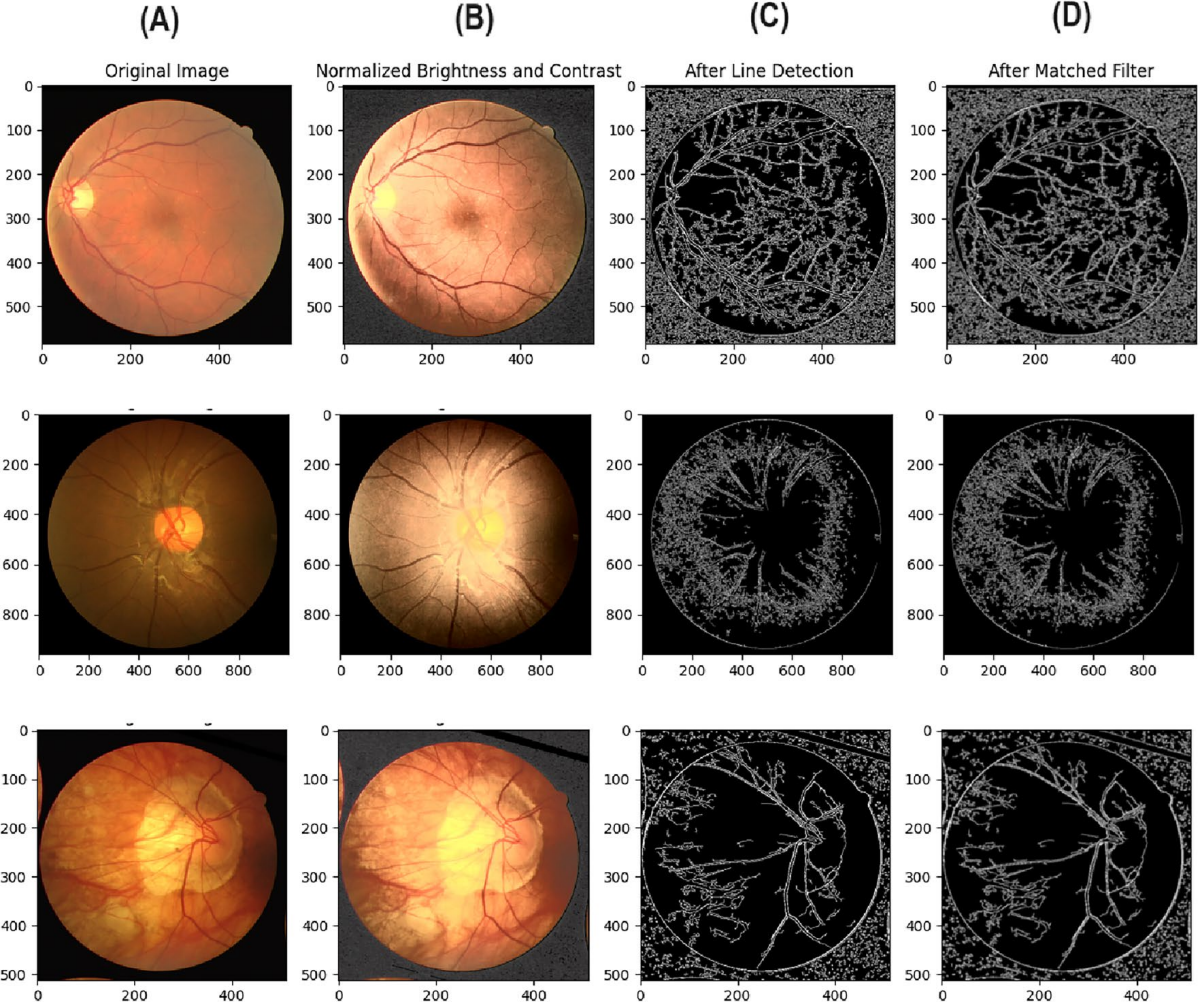
Illustration of the model preprocessing steps output result.

The proposed approach involves three distinct phases; each phase comprises various steps as described below:

- Preprocessing phase: This phase consists of five steps, each one associated with specific techniques to preprocess the retinal images and then use them in the segmentation phase, However, an illustration of the model preprocessing steps output result is shown in Fig. 2. The individual preprocessing steps are explained below:
  1. Initialize the environment parameters and load the medical images. Then, to ensure uniformity in feature range and have a common scale, each retinal image is preprocessed and normalized using Eq. (1).
    1 $$\begin{aligned} I_{norm} = \frac{I - \mu }{\sigma } \end{aligned}$$
    where $I$ is the original image, $\mu$ represent the mean pixels value, $\sigma$ is the standard deviation, and $I_{norm}$ represents the normalized image.
  2. Perform the color space conversion to extract relevant channels that contain blood vessels.
  3. Apply the matched filter technique, where the match filter bank is designed based on typical blood vessel profiles, such as line shapes of varying widths. The input image undergoes convolution with this filter, and the maximum response across the filter is computed for each pixel using Eqs. (2 and 3). To design a matched filter bank, let’s suppose $F = \{f_1, f_2, \ldots , f_N\}$ be a set of $N$ matched filters designed using blood vessel profiles. Convolve the input image using the filter bank; suppose $R(i, j, k)$ represents the response of the $k$-th filter for pixel $(i, j)$ in the convolved image as shown in Eq. (2).
    2 $$\begin{aligned} R(i, j, k) = I_{norm} * f_k(i, j) \end{aligned}$$
    where $*$ represent the convolution operation. Then, compute the maximum response $M(i, j)$ for pixel $(i, j)$ across the filter bank as shown in Eq. (3):
    3 $$\begin{aligned} M(i, j) = \max _k R(i, j, k) \end{aligned}$$
    where $k = 1$ to $N$. This process generates a “matched filter response” image, highlighting potential blood vessel locations.
  4. Integrate a multi-scale line detection technique, such as a Hessian-based method or a Steerable filter, as shown in Eq. (4). The detections from each scale are combined into a “line detection response” image, which further helps identify blood vessel locations. Let’s suppose $L(i, j, s)$ represent the line detection response at pixel $(i, j)$ for scale $s$.
    4 $$\begin{aligned} L(i, j, s) = \text {LineDetection}(I_{norm}, s) \end{aligned}$$
    where $\text {LineDetection}()$ denotes the line detection algorithm applied to the normalized image $I_{norm}$.
  5. Create the scale-space representation of the image according to Eq. (5), by generating a series of images that represent the original image at various scales. These representations are combined to highlight blood vessels, enhancing the visibility of their structures.
    5 $$\begin{aligned} S_m(i, j) = \text {ScaleSpace}(I_{norm}, m) \end{aligned}$$
    where $S = \{S_1, S_2, \ldots , S_M\}$ represents a set of $M$ scale-space images representing the original image at different scales, and $\text {ScaleSpace}()$ generates the $m$-th scale-space image for the normalized image $I_{norm}$.
- Segmentation phase: In this phase, we train the U-Net model with residual connections using the images generated from the prior steps, where the ground truth vessel segmentation is known. The U-Net model’s convolution operation helps the model recognize patterns and features indicative of blood vessels using Eq. (6). Incorporating the residual connections enhances the approach’s capacity to learn more complex features and representations of the data, including detecting small blood vessels.
  6 $$\begin{aligned} I'_{x,y} = \sum _{i=-k}^{k} \sum _{j=-k}^{k} I_{x+i,y+j} \cdot K_{i,j} \end{aligned}$$
  where $I'$ is the convolved image, $I$ is the original image, $K$ is the convolution kernel, and $k$ is the size of the kernel. After completing the U-Net training process, we evaluate the model’s performance on unseen images using several metrics, including accuracy, precision, F1 measure, Kappa, and others. This evaluation offers valuable insights into the model’s ability to identify and segment retinal blood vessels accurately.
- Postprocessing phase: In this phase, we use morphological techniques to enhance the output of the segmentation obtained from the U-Net model (${\textbf {V}}$), including removing noise from vessels or filling gaps to improve the quality and connectivity of the segmented vessels. Subsequently, the postprocessing stage involves applying a threshold to the final output to create a binary segmentation image, as shown in Eq. (7). This thresholding step transforms the segmented vessel probabilities into a binary map, classifying pixels as either vessel or non-vessel.
  7 $$\begin{aligned} B(i, j) = \text {Threshold}(V(i, j)) \end{aligned}$$
  where $\text {Threshold}()$ converts the pixel value $V(i, j)$ to a binary value based on a predefined threshold.

## Result analysis and experiment description

### Datasets

We evaluated the CoDLRBVS approach in terms of performance and effectiveness using several metrics over widely known benchmark datasets for blood vessel segmentation, such as DRIVE (Digital Retinal Images for Vessel Extraction) dataset^58^, CHASE_DB1 dataset^59^, High-Resolution Fundus (HRF) Image Database^2^, STARE (STructured Analysis of the Retina) dataset^60^, and the Retina Blood Vessel^61^, Table 1 provides more details about the used datasets, such as the number of training and testing samples, image height and width. However, the DRIVE database contains 20 retinal images captured using a Canon CR5 camera in a 24-bit color space. Each image comes with expert annotations of vascular segmentation, serving as ground truth for performance evaluation^58^. Similarly, the CHASE_DB1 dataset offers 28 high-resolution images of multi-ethnic children’s retinas, each with two sets of manual segmentations, providing a rich ground for algorithm testing and validation^59^. The HRF image database, known for its detailed imagery used in various comparative studies, is a valuable resource for algorithm evaluation^60^. With its 20 retinal fundus images, the STARE dataset provides additional variance and challenges, including images with and without pathology, crucial for assessing the adaptability and robustness of the segmentation algorithm^60^. Further, the 100 retinal images from^61^ offer additional challenges for retinal blood vessel segmentation, contributing significantly to developing and evaluating advanced segmentation algorithms^61^.

**Table 1:** Overview of the compared with retina vessel segmentation datasets.

Dataset	H $\times$ W	Imgs	Train	Test
DRIVE^58^	584 $\times$ 565	40	20	20
STARE^60^	605 $\times$ 700	20	10	10
CHASE_DB1^59^	960 $\times$ 999	28	20	8
Retina Blood Vessel^61^	584 $\times$ 565	120	100	20
HRF^2^	2336 $\times$ 3504	45	30	15

### Algorithm evaluation and discussion

The CoDLRBVS approach has been validated from different angles using various metrics across all earlier-mentioned datasets, such as (1) the Jaccard index (Eq. 8), which evaluates the similarity between the predicted segmentation and the ground truth to give a clear understanding of the overlap between segmented and actual vessels. (2) The F1 score (Eq. 9) harmonizes precision and recall, reflecting the accuracy and thoroughness of covering vessel pixels. (3) Sensitivity (Eq. 10) is vital for ensuring no vessel regions are missed, significant in diagnostic contexts. (4) Precision (Eq. 11) is critical in clinical settings to minimize false positives and avoid misdiagnosis. (5) Accuracy (Eq. 12) reflects overall correctness, while (6) the Kappa coefficient (Eq. 13) provides a normalized measure of agreement between the model prediction and the ground truth. (7) Area under the curve (AUC) is essential for understanding trade-offs at various thresholds. (8) Specificity (Eq. 14) measures correct identification of non-vessel areas, and finally, (9) average frames per second (FPS) (Eq. 15) indicates computational efficiency, which is essential for real-time applications. Therefore, these metrics provide a comprehensive evaluation framework for our approach.

8 $$\begin{aligned} J = \frac{|A \cap B|}{|A \cup B|} \end{aligned}$$

where $J$ is the Jaccard index, $A$ is the set of true positives, and $B$ is the set of predicted positives.

9 $$\begin{aligned} F1 = 2 \times \frac{\text {Precision} \times \text {Recall}}{\text {Precision} + \text {Recall}} \end{aligned}$$

where $F1$ is the F1 score, precision is the proportion of true positive predictions in all positive predictions, and recall is the proportion of true positive predictions in all actual positives.

10 $$\begin{aligned} \text {Recall} = \frac{TP}{TP + FN} \end{aligned}$$

where recall is the true positive rate, $TP$ is the number of true positives, and $FN$ is the number of false negatives.

11 $$\begin{aligned} \text {Precision} = \frac{TP}{TP + FP} \end{aligned}$$

where precision is the proportion of true positives in the predicted positive cases, $TP$ is the number of true positives, and $FP$ is the number of false positives.

12 $$\begin{aligned} \text {Acc.} = \frac{TP + TN}{TP + TN + FP + FN} \end{aligned}$$

where $Acc.$ is the accuracy of the model, $TP$ is the number of true positives, $TN$ is the number of true negatives, $FP$ is the number of false positives, and $FN$ is the number of false negatives.

13 $$\begin{aligned} \kappa = \frac{p_o - p_e}{1 - p_e} \end{aligned}$$

where $\kappa$ is the Kappa coefficient, $p_o$ is the relative observed agreement among raters, and $p_e$ is the hypothetical probability of chance agreement.

14 $$\begin{aligned} \text {Specificity} = \frac{TN}{TN + FP} \end{aligned}$$

where specificity is the true negative rate, $TN$ is the number of true negatives, and $FP$ is the number of false positives.

15 $$\begin{aligned} \text {FPS} = \frac{\text {Total Frames}}{\text {Total Time}} \end{aligned}$$

where $FPS$ is the average frames per second, Total Frames is the total number of frames or images processed, and Total Time is the total time taken for processing.

**Table 2:** CoDLRBVS model metric results over DRIVE, CHASE_DB, HRF, DRIVE retinal blood vessel, and STARE dataset.

Metric	DRIVE	CHASE_DB	HRF	Retinal blood vessel	STARE
Jaccard	0.6820	0.5276	0.4713	0.6717	0.7014
F1	0.8473	0.6903	0.6372	0.7763	0.8469
Recall	0.8048	0.5568	0.5035	0.7972	0.9708
Precision	0.9699	0.9094	0.8888	0.9301	0.9080
Accuracy	0.9629	0.9619	0.9581	0.9903	0.9655
Kappa	0.8248	0.6713	0.6170	0.8055	0.8290
AUC	0.8904	0.7761	0.7491	0.8849	0.9308
Sensitivity	0.9961	0.9954	0.9947	0.9927	0.9909
Specificity	0.8048	0.5568	0.5035	0.7972	0.9708
Average FPS	0.9669	0.8179	0.8279	0.8383	0.9246

**Figure 3:**
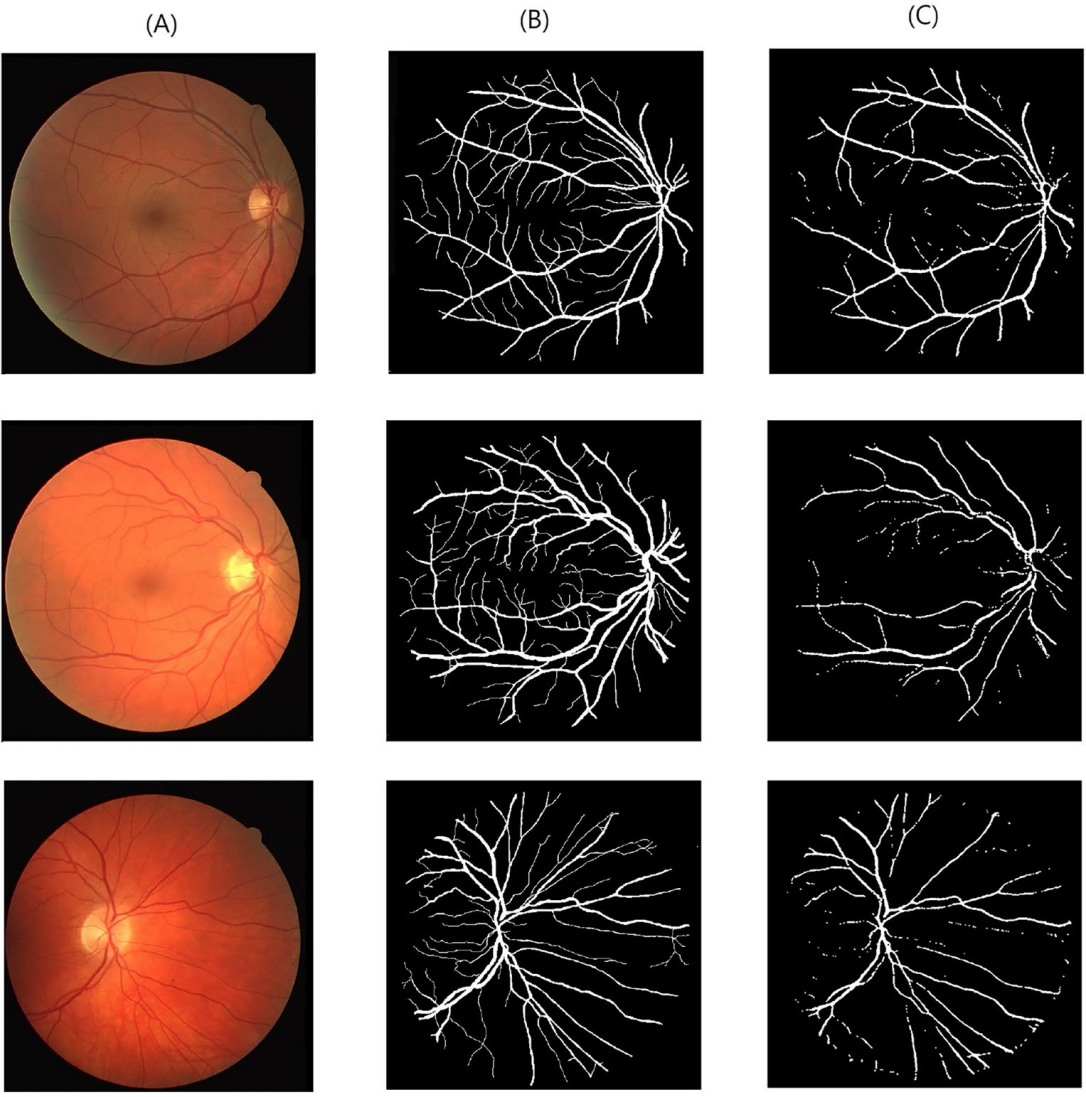
Illustration of retinal images (**A**), ground-truth (**B**) and output images after segmentation (**C**) over DRIVE dataset.

**Figure 4:**
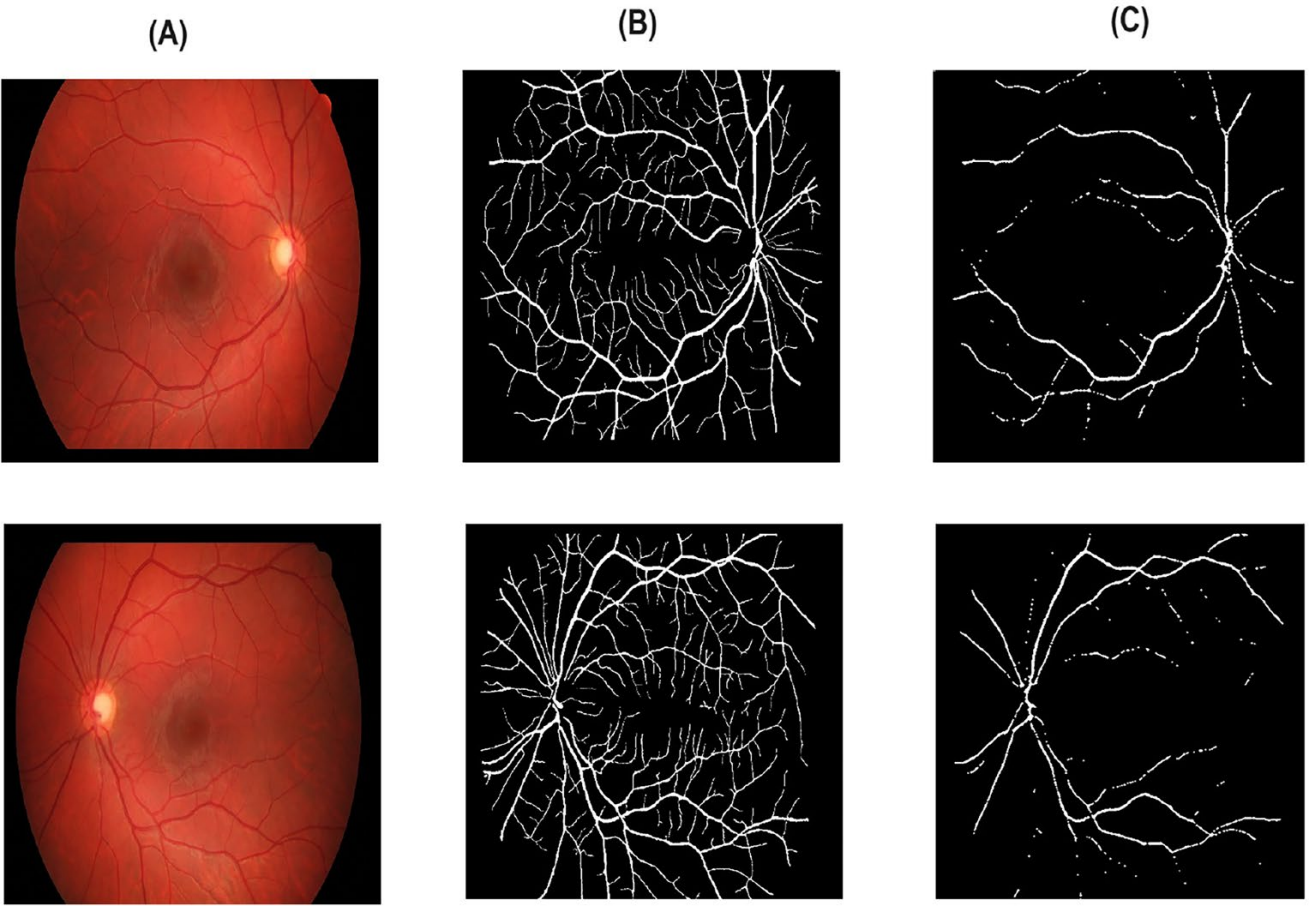
Illustration of retinal images (**A**), ground-truth (**B**) and output images after segmentation (**C**) over HRF dataset.

**Figure 5:**
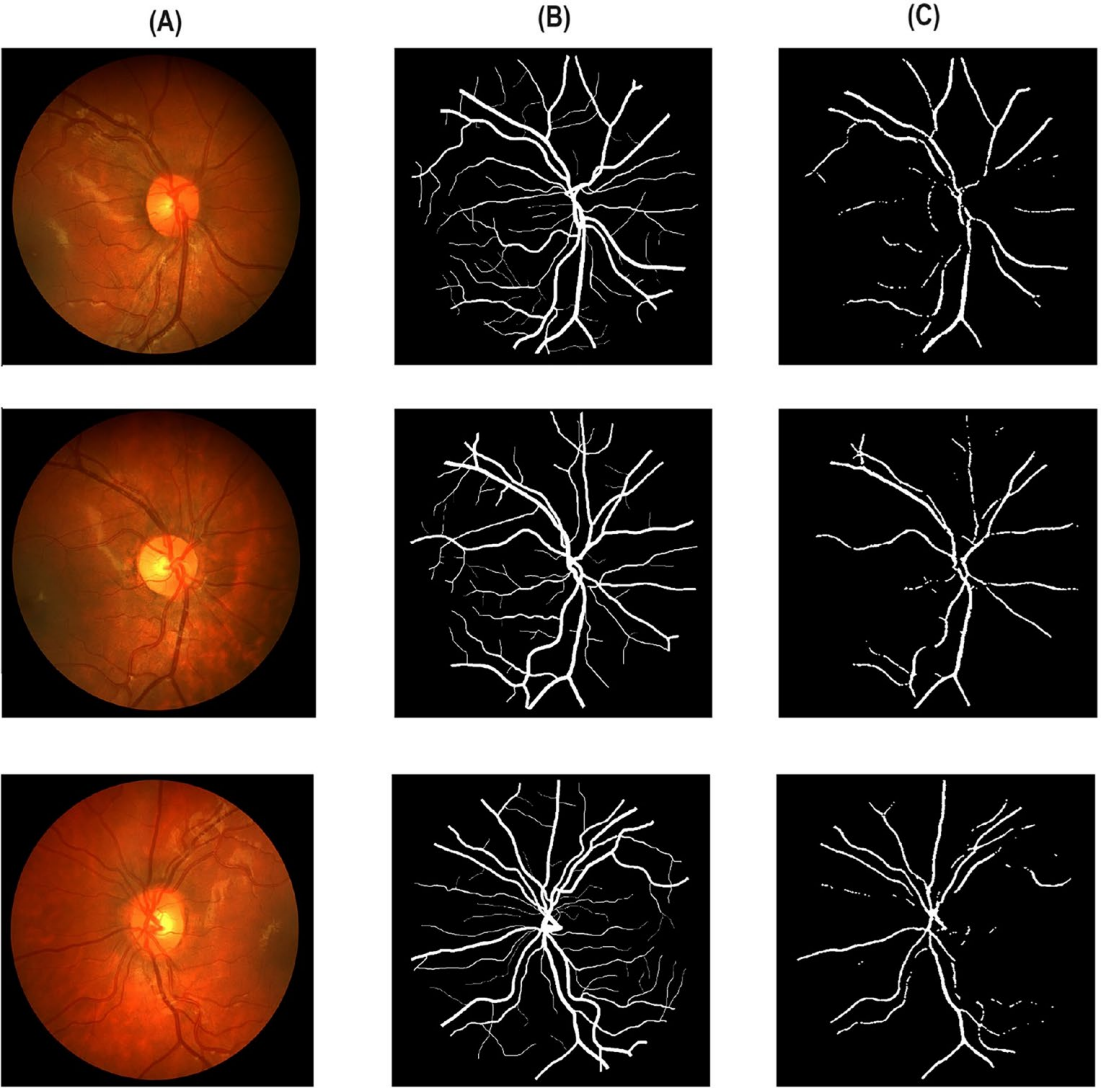
Illustration of retinal images (**A**), ground-truth (**B**) and output images after segmentation (**C**) over CHASE dataset.

**Figure 6:**
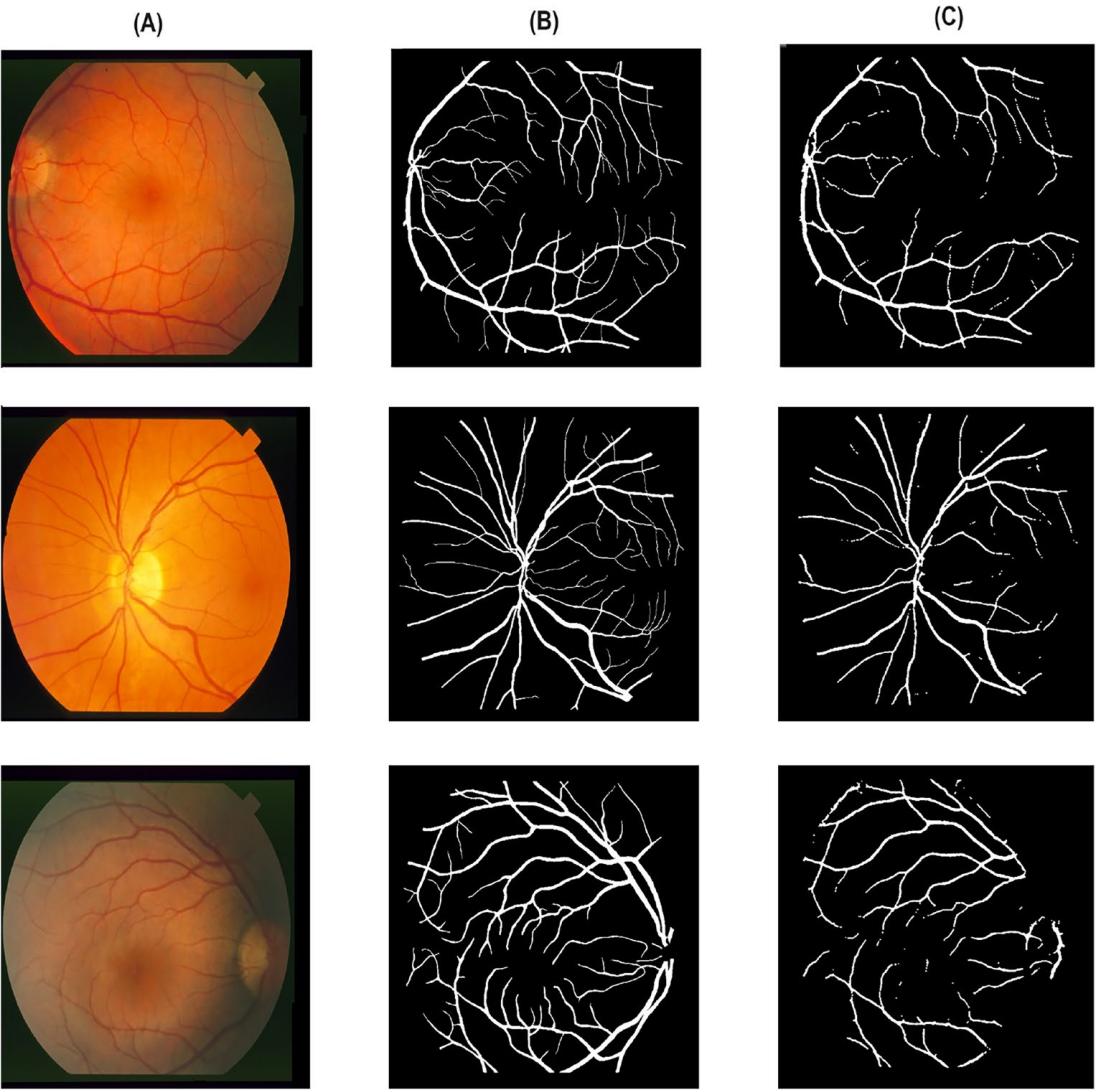
Illustration of retinal images (**A**), ground-truth (**B**) and output images after segmentation (**C**) over HRF dataset.

Table 2 reports different performance metrics used to evaluate the CoDLRBVS approach across various retinal image datasets, including DRIVE, CHASE, HRF, retinal blood vessel and STARE datasets. The results show that the proposed approach achieved a high accuracy of 0.9903% over the retinal blood vessel images dataset that contains the largest number of images, which is 100 images, and this is due to the fact that it was trained more than other datasets, and this indicates that training the model with more images results in more accurate results, further CoDLRBVS achieved an accuracy of 0.9629%, 0.9619%, 0.9581%, 0.9655% over DRIVE, CHASE, HRF, and STARE dataset respectively, underscore its effectiveness in accurately segmenting blood vessels. Such robustness across datasets suggests the CoDLRBVS’s generalizability and adaptability to different imaging conditions and vessel structures. The results also show that the model achieved very good Precision results over all datasets, where it achieved 0.9301% on the Retinal Blood Vessel and close to or exceeding 0.9% on the other datasets, including 0.9080% on STARE, highlight the approach proficiency in identifying true positive pixels across varied datasets, reducing false positives significantly. The Sensitivity of the model remains consistently high, with 0.9927%, 0.9961%, 0.9954%, 0.9947%, 0.9909% over the retinal blood vessel, DRIVE, CHASE, HRF, and STARE dataset respectively, indicating the CoDLRBVS’s ability to detect the majority of actual vessel pixels, a testament to the U-Net’s feature-learning capabilities and the effectiveness of the scale space representation. further the results in Table 2 show that the specificity has a wider variation with 0.7972% on the retinal blood vessel dataset, 0.8048% on DRIVE, lower values on CHASE_DB and HRF datasets, and a significantly higher 0.9708% on STARE, reflecting a challenge in distinguishing non-vessel regions across different datasets, which could be attributed to dataset-specific characteristics and noise factors. This metric’s variation underscores the need for dataset-specific adjustments to optimize the model’s performance. Moreover, CoDLRBVS achieves commendable F1 scores, indicating a balanced precision and recall, with the highest being 0.8473% on the DRIVE dataset and a close 0.8469% on STARE. The Jaccard index also demonstrates the model’s segmentation accuracy, with the highest value of 0.6820% on DRIVE and a comparable 0.7014% on STARE. The average frames per second (FPS) values, with the highest 0.966867% on DRIVE and 0.924556% on STARE, indicate the model’s efficiency, suggesting its potential for real-time applications. In addition, the integration of matched filter, multi-scale line detection, and U-Net, along with strategic post-processing, offers a comprehensive approach that caters to the nuances of retinal blood vessel segmentation. Figures 3, 4 , 5 and 6, shows the results of the vessel segmentation method from studied datasets. In all figures; (A) shows the original RGB image; (B) shows the ground truth images, which are manually segmented by experts; (C) shows the vessel segmentation results of the proposed model, highlighting the algorithm’s performance in accurately delineating blood vessels.

As it can be seen in Figs. 3 , 4, 5 and  6, that the resulted images (c) serves as a testament to the model’s proficiency in segmenting retinal blood vessels, exhibiting a high degree of detail with both central and peripheral vessels crisply outlined against the contrasting background. The continuity and uniformity of the vessel structures, mirroring the ground truth with notable precision, highlight the model’s capability to capture essential details necessary for accurate segmentation.

**Table 3:** Performance of multiple vessel segmentation methods using the DRIVE dataset.

Method	Sensitivity	Accuracy	Specificity
Niemeijer et al.^62^	0.6898	0.9417	0.9696
Martinez-Perez et al.^63^	0.7246	0.9344	0.9655
Ramlugun et al.^64^	0.6413	0.9341	0.9767
Fraz et al.^4^	0.7152	0.9430	0.9768
Soares et al.^40^	0.7230	0.9446	0.9762
You et al.^65^	0.7410	0.9434	0.9751
Marin et al.^66^	0.7067	0.9452	0.9801
Yali Zhao et al.^67^	0.7359	0.9418	0.9720
Staal et al.^41^	0.7194	0.9442	0.9773
Mendonca et al.^68^	0.7344	0.9452	0.9764
Zhang et al.^69^	0.7120	0.9382	0.9724
Li et al.^70^	0.7154	0.9343	0.9716
Fraz et al.^46^	0.7406	0.9480	0.9807
Ricci et al.^71^	–	0.9595	–
Proposed CoDLRBVS	0.9961	0.9629	0.8048

**Table 4:** Comparison with existing retinal vessel segmentation methods using STARE dataset.

Method	Sensitivity	Accuracy	Specificity
Martinez-Perez et al.^63^	0.7506	0.9410	0.9569
Fraz et al.^4^	0.7311	0.9442	0.9681
Marin et al.^66^	0.6944	0.9526	0.9819
Mendonca et al.^68^	0.6996	0.9440	0.9730
Staal et al.^41^	0.6970	0.9516	0.9810
You et al.^65^	0.7260	0.9497	0.9756
Hoover et al.^60^	0.6751	0.9267	0.9567
Yali Zhao et al.^67^	0.7769	0.9364	0.9550
Zhang et al.^69^	0.7171	0.9483	0.9753
Li et al.^70^	0.7191	0.9407	0.9687
Soares et al.^40^	0.7103	0.9480	0.9737
Fraz et al.^46^	0.7548	0.9534	0.9763
Ricci et al.^71^	–	0.9584	–
Proposed CoDLRBVS	0.9909	0.9655	0.9708

**Table 5:** Comparison with existing retinal blood vessel segmentation methods using CHASE database.

Method	Sensitivity (SE)	Accuracy (ACC)	Specificity (SP)
Human observer	0.74	–	0.97
Orlando et al.^47^	0.72	–	0.97
Karn et al.^72^	0.78	0.97	0.97
Zhang et al.^73^	0.77	0.96	0.98
Fraz et al.^74^	0.72	0.95	0.97
Roychowdhury et al.^75^	0.75	0.94	0.96
Fraz et al.^12^	0.72	0.94	0.97
Roychowdhury et al.^76^	0.72	0.95	0.98
Azzopardi et al.^77^	0.72	0.94	0.96
Chakraborti et al.^78^	0.53	0.93	0.95
Fan et al.^79^	0.65	0.95	0.97
Biswal et al.^80^	0.76	–	0.97
Proposed CoDLRBVS	0.9954	0.9619	0.5568

**Table 6:** Blood vessel segmentation comparison on HRF database.

Method	Sensitivity	Accuracy	Specificity
Jiang et al.^81^^∗^	0.8010	0.9650	0.8010
Joshua^82^	0.8059	0.9688	0.9826
Orlando et al.^47^	0.7874	–	0.9584
Jiang et al.^81^^∗∗^	0.7686	0.9662	**0.9826**
Zhou et al.^83^	0.8015	0.9544	0.9699
Odstrcilik et al.^84^	0.7794	–	0.9650
Proposed CoDLRBVS	0.9947	0.9581	0.5035

Significant values are in [bold].

*Performance based on single database.

**Performance based on cross database.

As it can be seen in Table 3, which illustrates the comparison results of CoDLRBVS performance with different vessel segmentation methods using the DRIVE dataset. The proposed CoDLRBVS model demonstrates high sensitivity, with a score of 0.9961%, indicating its outperforming capability in correctly identifying retinal blood vessels. This metric is significantly higher than the other listed methods, which range between 0.64 and 0.74%, suggesting that CoDLRBVS is particularly effective at minimizing false negatives and reliably detecting even the most delicate vessels. However, while its accuracy is competitive at 0.9629%, indicating a high overall rate of correct predictions, its specificity is comparatively lower at 0.8048%. This lower specificity implies a higher rate of false positives, meaning the model might sometimes mistakenly identify non-vessel areas as vessels.

As it can be seen in Table 4, which displays the performance of the CoDLRBVS with other compared algorithms over the STARE dataset, CoDLRBVS also exhibits outstanding sensitivity at 0.9909%, significantly higher than other methods, which range from approximately 0.6751 to 0.7769%. Furthermore, in terms of accuracy, CoDLRBVS performs well with a score of 0.9655%, suggesting that it generally makes correct predictions. This indicates that the model has reliable performance in segmenting retinal blood vessels. The model’s specificity is also high at 0.9708%, denoting its ability to identify non-vessel areas over the STARE dataset correctly. This specificity is competitively placed within the range of other methods, which mostly fall between 0.9550 and 0.9819%. When comparing methods across the board, it’s evident that while many provide balanced performance, CoDLRBVS’s standout feature remains its exceptional sensitivity, making it a potentially valuable tool for medical imaging tasks where missing a small detail can lead to significant consequences.

The results in Tables 5 and 6 show that the CoDLRBVS model exhibits a very high sensitivity of 0.9954% and 0.9947% in both CHASE DB1 and HRF datasets respectively, whereas in term of accuracy, the CoDLRBVS model achieved 0.9619% and 0.9581% which is considered comparable with other models, where its values range from 0.94 to 0.97%. However, combining matched filters, multi-scale line detection, u-net architecture, scale space, and morphological techniques has contributed to robust feature recognition and precise vessel detection in CoDLRBVS, ensuring minimal false negatives. However, the CoDLRBVS model’s specificity is notably lower than other methods. While the model excels at detecting vessels (high sensitivity), it also tends to mark non-vessel elements as vessels (lower specificity). This could be due to the nature of the dataset.

The proposed model, however, maintains high performance, demonstrating a balanced and robust approach to retinal vessel segmentation. This balanced performance is due to the integration of deep learning U net with various techniques, each contributing to different aspects of the segmentation of retinal blood vessels. whereas, the matched filter and multi-scale line detection techniques are used in the pre-processing stages of the model. The matched filter is designed to respond maximally to typical blood vessel profiles, enhancing the image’s vessels. multi-scale line detection, on the other hand, identifies vessels of varying widths across the image. Combining these two methods benefits the model due to the fact that both methods facilitate model sensitivity to the presence of vessels and adaptivity to their varying widths, where the single-scale or non-adaptive methods might miss.

Further, applying the U-Net architecture, a deep learning model, plays a crucial role in segmenting the blood vessels. Its ability to learn and extract high-level features from the retinal images contributes to the high sensitivity of the CoDLRBVS hybrid model. The U-Net architecture, with its encoding and decoding pathways, is designed specifically for segmentation tasks, and it utilizes the spatial information in the image, which is crucial for precise segmentation. The U-Net’s ability to generalize and learn complex representations likely contributes to the CoDLRBVS hybrid model’s superior accuracy results.

Furthermore, the scale space representation technique helps handle the varying size of retinal blood vessels. It generates images representing the original image at different scales, which is particularly beneficial for capturing both larger vessels and smaller, more intricate vascular structures. This likely contributes to the model’s balanced performance in terms of sensitivity and specificity, as it helps ensure that vessels of all sizes are accounted for in the segmentation. Finally, the morphological techniques are applied in the post-processing stage to refine the segmentation. Morphological operations can remove noise (small false-positive detections) and fill in gaps in detected vessels (false negatives), leading to a cleaner and more accurate final segmentation. It is worth noting that it would be interesting to extend our research on a federated learning scale^85–87^.

## Conclusion

This paper presents CoDLRBVS, a pioneering cognitive-based deep learning model for medical image processing, namely retinal blood vessel segmentation. Our approach combines (1) a Matched Filter to detect segmentation in noisy data. It works by designing a filter that matches the shape of the signal being transmitted or received. (2) Multi-scale line detection, a technique to capture the vessels at different angles at that point. (3) U-Net architecture, a deep learning semantic segmentation technique. (4) Scale space for handling images at different scales, suppressing fine-scale structures. Moreover, (5) morphological techniques extract features based on an image’s topographic surface. Our model demonstrates a remarkable balance across performance metrics, achieving notable mean accuracy of 96.7%, precision of 96.9%, sensitivity of 99.3%, and specificity of 80.4% across all of the studied datasets, positioning it as a strong contender among existing state-of-the-art methods. The model’s high specificity significantly mitigates false positives, which is vital for precise segmentation. Hence, the novelty of our approach lies in its balanced performance, avoiding the common trade-offs among sensitivity, specificity, and accuracy observed in other methods. This balance reflects the model’s robustness and the efficacy of integrating various techniques following the cognitive computing principles. Hence, CoDLRBVS improves the accuracy and efficiency of retinal image analysis and enhances the ability to detect and diagnose retinal diseases. Future work will broaden dataset testing to enhance our model’s versatility and incorporate cutting-edge techniques and architectures to refine its performance.

## Data Availability

The data used in this study are available on the following websites: https://paperswithcode.com/dataset/drivehttps://www.kaggle.com/datasets/abdallahwagih/retina-blood-vesselhttps://www.kaggle.com/datasets/pradosh123/retinal-vessel-segmentation-combinedhttps://www.kaggle.com/datasets/rashasarhanalharthi/chase-db1.
